# A SAS-6-Like Protein Suggests that the Toxoplasma Conoid Complex Evolved from Flagellar Components

**DOI:** 10.1128/EC.00096-13

**Published:** 2013-07

**Authors:** Jessica Cruz de Leon, Nicole Scheumann, Wandy Beatty, Josh R. Beck, Johnson Q. Tran, Candace Yau, Peter J. Bradley, Keith Gull, Bill Wickstead, Naomi S. Morrissette

**Affiliations:** Department of Molecular Biology and Biochemistry, University of California, Irvine, Irvine, California, USAa; Sir William Dunn School of Pathology, University of Oxford, Oxford, United Kingdomb; Department of Molecular Microbiology, Washington University School of Medicine, St. Louis, Missouri, USAc; Department of Microbiology, Immunology and Molecular Genetics, University of California, Los Angeles, Los Angeles, California, USAd; Centre for Genetics and Genomics, University of Nottingham, Nottingham, United Kingdome

## Abstract

SAS-6 is required for centriole biogenesis in diverse eukaryotes. Here, we describe a novel family of SAS-6-like (SAS6L) proteins that share an N-terminal domain with SAS-6 but lack coiled-coil tails. SAS6L proteins are found in a subset of eukaryotes that contain SAS-6, including diverse protozoa and green algae. In the apicomplexan parasite Toxoplasma gondii, SAS-6 localizes to the centriole but SAS6L is found above the conoid, an enigmatic tubulin-containing structure found at the apex of a subset of alveolate organisms. Loss of SAS6L causes reduced fitness in Toxoplasma. The Trypanosoma brucei homolog of SAS6L localizes to the basal-plate region, the site in the axoneme where the central-pair microtubules are nucleated. When endogenous SAS6L is overexpressed in Toxoplasma tachyzoites or Trypanosoma trypomastigotes, it forms prominent filaments that extend through the cell cytoplasm, indicating that it retains a capacity to form higher-order structures despite lacking a coiled-coil domain. We conclude that although SAS6L proteins share a conserved domain with SAS-6, they are a functionally distinct family that predates the last common ancestor of eukaryotes. Moreover, the distinct localization of the SAS6L protein in Trypanosoma and Toxoplasma adds weight to the hypothesis that the conoid complex evolved from flagellar components.

## INTRODUCTION

Centrioles and basal bodies are microtubule-based structures found in many eukaryotic lineages, including animals, lower plants, and diverse unicellular organisms (1–3). Basal bodies are distinguished from centrioles by association with a flagellar axoneme that is templated from an extension to the centriole known as a transition zone (1, 4). The widespread occurrence of centrioles and basal bodies shows that they have an ancient origin in eukaryotes (5–8), but lineages such as flowering plants and most fungi have lost the ability to build these structures. Centrioles and basal bodies typically contain nine triplet microtubules organized with radial symmetry, although they can rarely be built of nine doublet or singlet microtubules (e.g., those in Drosophila embryos and Caenorhabditis elegans testes, respectively) or have likely 6-fold symmetry (9, 10). In spite of their presence at spindle poles, centrioles are dispensable for bipolar spindle formation in several lineages (11–15). However, all species that build centrioles/basal bodies have flagella at some stage of their life cycle; this observation likely underlies the true evolutionary imperative of these structures (2, 3).

Many protozoan organisms, including apicomplexan and kinetoplastid parasites, use spatially separated and morphologically distinct microtubule-organizing centers (MTOCs) to organize individual microtubule populations (16). Kinetoplastid parasites of the genera Trypanosoma and Leishmania cause human infectious diseases, including African sleeping sickness, Chagas disease, and kala azar (17). The shape of these organisms is maintained by an array of ∼100 densely packed corset microtubules that underlie the plasma membrane (18). The single flagellum is nucleated by a membrane-docked basal body that is distant from the nucleus (18–22). Flagella are not disassembled during division, and in replicating cells, basal bodies do not contribute to the organization of the poles of the intranuclear spindle (23). Apicomplexan parasites also cause a variety of medically significant diseases, including malaria, toxoplasmosis, and cryptosporidiosis (17). Apicomplexans typically have a complex life cycle that involves both asexual and sexual replication, and they alter microtubule architecture between the sexual and asexual life cycle stages. Microgamete motility is required for fertilization of macrogametes and is powered by flagella that originate from apical basal bodies (24). Asexual stages lack flagella and use a characteristic actin-and-myosin-based gliding motility to invade host cells (25, 26). These asexual forms (e.g., merozoites, tachyzoites) have two microtubule populations; spindle microtubules coordinate chromosome segregation during mitosis, and subpellicular microtubules subtend the pellicle to impose an elongated cell shape and cell polarity (27–29). Each microtubule population is associated with a distinct MTOC; subpellicular microtubules radiate from the apical polar ring (APR), an MTOC unique to apicomplexan organisms (28, 30, 31), whereas spindle microtubules originate near a specialized region of the nuclear envelope termed the centrocone (32–35).

Toxoplasma gondii is a member of the Coccidia—a subclass of apicomplexan parasites that build two tubulin-based structures in addition to the spindle and subpellicular microtubules, i.e., the conoid and centrioles. The conoid is an apical organelle constructed of comma-shaped tubulin sheets that spiral to form a cone-shaped structure (36–38). Two preconoidal rings surmount the conoid, and when extended, the conoid and preconoidal ring complex reside above the APR (28, 39). The conoid and preconoidal rings can also retract through the APR to be surrounded by the subpellicular microtubules. The conoid is permanently retracted in intracellular parasites, but extracellular tachyzoites extend and retract this structure, a probing behavior that is believed to facilitate host cell invasion (36–38). Two short, closely apposed microtubules are located at the center of the conoid. In contrast to the apical conoid, Toxoplasma centrioles are found in the cytoplasm proximal to the nucleus and duplicated centrioles are found at the spindle poles in the region of the centrocone structures. Coccidian centrioles are composed of nine singlet microtubules and a central tubule and are organized in a parallel rather than an orthogonal configuration (27, 33, 40).

SAS-6 is required for centriole biogenesis in eukaryotes ranging from protozoa to vertebrates (41–44). Chlamydomonas reinhardtii mutants deficient in SAS-6 (*bld12*) produce basal bodies with variable numbers of triplet blades (45). Studies of nematodes and Drosophila indicate that SAS-6 is needed to form the “cartwheel hub” in nascent centrioles (45–49). The structural properties of SAS-6 elegantly dovetail with its ability to template a centriole (50, 51). SAS-6 assembles into homodimers that contain a globular head domain at the N terminus and an extended coiled-coil rod. Remarkably, SAS-6 homodimers can self-assemble into a structure with a central hub and nine rods, akin to the cartwheel observed in structural studies of centrioles. In this paper, we describe for the first time the properties of a family of SAS-6-like proteins that are related to SAS-6 but structurally distinct. We investigate the function of SAS-6-like proteins in two distantly related protozoa and discuss their evolutionary relationship to conventional SAS-6.

## MATERIALS AND METHODS

### Culture of parasites.

Toxoplasma lines, including the previously described RNG1-yellow fluorescent protein (YFP) line (31, 52), were grown in confluent monolayers of human foreskin fibroblast (HFF) cells as previously described (53). Trypanosoma brucei procyclic forms were cultured at 28°C in SDM-79 medium supplemented with 10% fetal calf serum (54).

### Generation of fusion protein lines.

To generate in-frame C-terminal chimeras with proteins of interest, regions of TGGT1_040920 (TgSAS-6), TGGT1_059860 (TgSAS6L), Tb927.10.7920 (TbSAS6L), and Tb11.02.5550 (TbWDR16) were amplified and cloned into LIC (52), pLew-MH-TAP-eYFP (55), and pEnT6P-Y (56) vectors by standard methods. TgSAS-6 and TgSAS6L constructs in the pYFP.LIC.DHFR vector were transfected into *ku80*-null Toxoplasma parasites as previously described, and stable lines were isolated by selection in 1 μM pyrimethamine and single cell cloned (52). The coding sequence of TgSAS6L was validated by amplification of cDNA encoding the complete open reading frame with primers described in Table 1. A pre-existing GRASP55-YFP construct (57) was modified to replace the GRASP55 coding sequence with SAS6L to drive the expression of TgSAS6L-YFP from the α1-tubulin promoter, and stable lines were isolated by selection for chloramphenicol resistance. Linearized plasmid DNA was used to transfect exponentially growing cultures of procyclic-form T. brucei by electroporation (three 100-μs pulses of 4.25 kV/cm). Transfected cells were selected by the addition of 1 μg/ml puromycin (pEnT6P-TbSAS6L1-Y) or 10 μg/ml blasticidin (pEnT6B-WDR16-mCherry). Inducible overexpression of TbSAS6L was achieved by cloning TbSAS6L into the pLew-MH-TAP-eYFP vector with primers described in Table 1. Transfectants were selected in 5 μg/ml phleomycin, and overexpression of TbSAS6L1-YFP was induced by the addition of 1 μg/ml doxycycline to the culture medium.

**Table 1:** Primer pairs used for molecular biology constructs in this study

Primer	Sequence
TbSAS6L CDS pEnT6P 5′	GTTGCGGCCGCAAAGAACCCCAACGCTTTTT
TbSAS6L CDS pEnT6P 3′	GTCTACTAGTTCGCGTTTTTCCAAGTGTG
TbSAS6L UTR pEnT6P 5′	GTTAAGCTTGAGTGCAAATTCTTCAGTGCG
TbSAS6L UTR pEnT6P 3′	GTTGCGGCCGCCGACATTAGCTCCGGTTTCT
WDR16 CDS pEnT6P 5′	CGTTCTAGAGCAGCGAAGGAGTACGAAA
WDR16 CDS pEnT6P 3′	TCGAGCACGGCCAGGTAGCTGAT
WDR16 UTR pEnT6P 5′	GCTCGAGCTTTCGCAAACCCATTCG
WDR16 UTR pEnT6P 3′	GTAGGATCCAACGGAAGGGTGGCAGTT
5′ TbSAS6L HindIII pLew	ATCAAGCTTATGGATCGCATAGAAATATACTACCAG
3′ TbSAS6L1 XhoI pLew	GATCTCGAGTCGCGTTTTTCCAAGTGTGACAGTAGC
SAS6L LIC vector 5′	TACTTCCAATCCAATTTAATGCACAGACGGAAATGCTCTCC
SAS6L LIC vector 3′	TCCTCCACTTCCAATTTTAGCGAGGAACCGAGTGGATGC
SAS6L MBP plasmid 5′	ATGGCGACAAACTTCGGCTTTGG
SAS6L MBP plasmid 3′	TATAAAGCTTTCAGAGGAACCGAGTGGATGCGCC
ptub-SAS6L-YFP 5′	CAAAGATCTATGGCGACAAACTTCGGCTTTGG
ptub-SAS6L-YFP 3′	TATACCTAGGGAGGAACCGAGTGGATGCGCC
SAS6L KO 5′ flank 5′	AAGGTACCCCCTCTCTTCACAGTCGAAGACC
SAS6L KO 5′ flank 3′	TTGGGCCCGTTATTCTGTTCGAACCCGGGG
SAS6L KO 3′ flank 5′	AAACTAGTCGGTGGGAGGTCTCAAGCG
SAS6L KO 3′ flank 3′	GCGCGGCCGCCACGTAATCACACAATCCGAGCGTATAG

### Toxoplasma SAS6L gene knockout.

We constructed a knockout vector with the pmini.GFP.HPT plasmid (58). A 4.9-kb region upstream of the TGGT1_059860 coding sequence and a 5-kb downstream region were amplified with primers listed in Table 1. These inserts were cloned upstream and downstream of the hypoxanthine xanthine guanine phosphoribosyl transferase (HPT) gene. We electroporated *ku80*Δ *hpt* mutant RH tachyzoites with the construct and selected for transformants in 50 μg/ml mycophenolic acid and 50 μg/ml xanthine. Single-knockout clones were isolated from two independent transfections by single-cell cloning. We also isolated green fluorescent protein (GFP)-expressing transformants with a nonhomologous integration of the knockout vector. These were used in competition assays with the knockout line (see below).

### Escherichia coli expression and purification of TgSAS6L.

The cDNA sequence of TgSAS6L was inserted into the c2x pMAL vector (NEB) in order to express a maltose-binding protein (MBP)-TgSAS6L fusion in E. coli. After induction for 2 h with 300 μM isopropyl-β-d-thiogalactopyranoside (IPTG), the protein was purified on an amylose column and cleaved away from MBP with Factor Xa. Dialyzed TgSAS6L was used as an immunogen to generate mouse polyclonal antiserum.

### Toxoplasma conoid extrusion.

Conoid extrusion was induced in freshly lysed extracellular parasites by treatment with HEPES-buffered saline supplemented with 5 μM ionomycin (Sigma) and 5 mM CaCl_2_ (Sigma) (36–38), and samples were fixed as previously described (31).

### Immunofluorescence staining and fluorescence microscopy.

YFP- or mCherry-tagged intracellular Toxoplasma parasites were fixed, permeabilized, and stained as previously described (27). Extracellular parasites were filtered through a 3-μm polycarbonate filter (GE Water & Process Technologies), centrifuged at 1,000 × *g* for 20 min at 4°C, and suspended in a small volume of phosphate-buffered saline (PBS). Detergent-extracted parasites were generated and stained as previously described (31). The antibodies used for immunofluorescence assays included a mouse polyclonal serum against TgSAS6L, a mouse monoclonal antibody against GFP (Roche), and a rabbit Toxoplasma-specific tubulin serum (27) detected with Alexa 594-, Alexa 488-, and Cascade blue-conjugated secondary antibodies (Invitrogen). DNA was visualized by 4′,6-diamidino-2-phenylindole (DAPI) staining. Toxoplasma samples were imaged on a Zeiss Axiovert 200M microscope with the AxioVision system. Trypanosomes were settled onto glass slides and either fixed with 1% formaldehyde for whole-cell preparations or extracted with 1% Nonidet P-40 in PEME [100 mM 100 mM piperazine-*N*,*N*′-bis(2-ethanesulfonic acid) (PIPES, pH 6.9), 2 mM EGTA, 1 mM MgSO_4_, 0.1 mM EDTA] for cytoskeleton preparations. Cells were labeled with BBA4 or YL1/2 antibodies, which were visualized with anti-mouse IgM–Cy5 and anti-rat IgG–tetramethyl rhodamine isothiocyanate (Jackson ImmunoResearch). Cells were mounted in 90% glycerol–50 mM sodium phosphate (pH 8) supplemented with 1% 1,4-diazabicyclo[2.2.2]octane and 0.4 μg ml^−1^ DAPI.

### Immunoblot assays.

Protein lysates containing ∼5 × 10^6^ Toxoplasma tachyzoites per lane were resolved by 15% SDS-acrylamide gel electrophoresis and transferred to nitrocellulose. After blocking for 1 h in 5% milk in PBS-Tween, the blot was probed with primary antibodies (1:300 anti-TgSAS6L and 1:750 anti-ISP3 in 5% milk) for 1 h, washed in PBS-Tween, probed with a secondary antibody (1:2,000 horseradish peroxidase-conjugated goat anti-mouse antibody; Invitrogen) for 1 h, and washed in PBS-Tween prior to detection by chemiluminescence assay.

### Electron microscopy (EM).

Freshly lysed Toxoplasma tachyzoites from stable TgSAS6L-YFP lines were isolated by filtration. Parasites were fixed and cryosectioned as previously described (31), with an anti-GFP antibody (Abcam). Trypanosomes were settled onto Formvar-coated nickel finder grids and extracted with 1% NP-40–PEME. For Ca^2+^ preparations, cells were additionally incubated with 65 mM Ca^2+^–PIPES, pH 6.9. Preparations were fixed with 2.5% glutaraldehyde–PEME for 10 min and then neutralized with 1% glycine–PEME. Grids were mounted in H_2_O, and native fluorescence was analyzed with a Leica DM5500 microscope. Grids were negatively staining with 1% aurothioglucose (USP) and viewed on an FEI Tecnai-F12 electron microscope.

### Toxoplasma competition assays.

T_25_ flasks with confluent HFF cells were inoculated with a 1:1 ratio of *sas6l*-null parasites and GFP-expressing clones from the same transfection that harbor a nonhomologous integration of the knockout vector (10^7^ parasites of each line) as described in a previously established competition assay (59). The relative numbers of GFP-positive (control) and GFP-negative (*sas6l*-null) parasites were determined by flow cytometry (Apogee Flow Systems).

### Alignments and phylogenetic analysis.

SAS6 and SAS-6-like proteins form a natural set on the basis of a BLASTp clustering approach (60). This clustering results from the presence of a conserved domain of ∼200 aa covered by a Pfam-B domain (Pfam_B_2528) of unknown function. To define the family of SAS6 and SAS-6-like proteins, the hidden Markov model defining Pfam_B_2528 was used to search the complete predicted proteomes of 44 diverse eukaryotes (HMMER3.0; hmmer.janelia.org). All 69 proteins containing the domain (e-value, <0.001) were extracted, trimmed to the conserved domain, and aligned by MAFFT v6.811b by adopting the E-INS-i strategy (61). Bayesian phylogenies were inferred from four runs of the metropolis-coupled Markov chain Monte Carlo method implemented in the program MrBayes3.1.2 (62) (WAG substitution matrix; GTR+Γ+I with Γ distribution approximated to four discrete categories and shape parameters estimated from the data). Maximum-likelihood support for the inferred topology was generated from 100 bootstrap replicates of the data with PhyML3.0 (63) (LG matrix; GTR+Γ+I, as described above).

## RESULTS

### SAS-6 and SAS6L localize to distinct structures in Toxoplasma tachyzoites.

**Fig 1:**
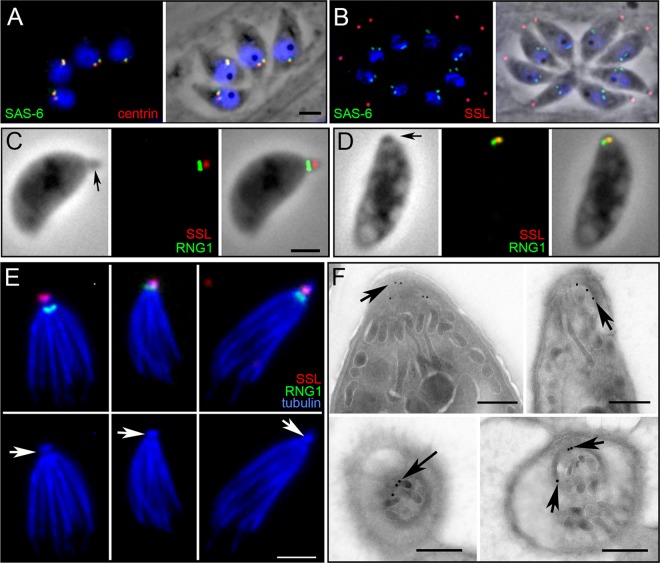
TgSAS6 and TgSAS6L localize to distinct regions in Toxoplasma tachyzoites. (A) The Toxoplasma homolog of SAS6 (green) localizes to centrioles along with the centriole marker centrin (red). The overlap between these proteins is not complete, which likely reflects distinct localization within centrioles; while centrin is typically located throughout the centriole, SAS-6 is a marker of the proximal end of centrioles. (B) TgSAS6L (red, anti-SAS6L antiserum) is found at the apex of Toxoplasma tachyzoites and does not colocalize with SAS-6 in the centrioles (green, YFP tag). (C) Ionomycin triggers conoid extrusion in most extracellular parasites. In these cells, SAS6L-mCherry labeling (red) is above the APR (RNG1-YFP, green). (D) When the conoid is retracted, TgSAS6L and RNG1-YFP partially colocalize (the preconoidal region is smaller than the APR). (E) Extraction of extracellular tachyzoites with DOC frees the subpellicular microtubules and conoid (blue) and APR (RNG1-YFP, green) from other cellular material. TgSAS6L is located at the apical end of the conoid, and RNG1-YFP is beneath the extended conoid at the APR. The tubulin channel alone permits identification of the conoid (arrows) above the subpellicular microtubules. (F) Immunogold labeling indicates that SAS6L-YFP localizes to the extreme apical region of Toxoplasma tachyzoites, above the conoid in both longitudinal (arrows, top) and transverse (arrows, bottom) sections of the apical region. Scale bars, 2 μm (A to E) or 250 nm (F).

SAS-6 is an essential and universal component of centrioles that creates the “cartwheel hub” found at the proximal end of the centriole barrel (41, 42, 45). We came upon the hypothetical protein TGGT1_059860 during a genome database search (www.ToxoDB.org) for the Toxoplasma homolog of SAS-6. Since TGGT1_059860 has similarity to SAS-6, we named it SAS-6-like (SAS6L). When the Toxoplasma SAS-6 homolog (TGGT1_040920) was tagged by fusion of the C terminus to YFP, the label localized exclusively to the centriole, coincident with the centriolar marker centrin (Fig. 1A), as is expected from the localization of SAS-6 in other systems. In contrast, TgSAS6L localizes exclusively to a region at the apex of the parasites (Fig. 1B). Since TgSAS6L labeled a small spot in the apex of Toxoplasma tachyzoites, we induced conoid extrusion in extracellular parasites and used a marker for the APR (RNG1) (31) to localize TgSAS6L more accurately. TgSAS6L is found at the apical tip of extended conoids, above the RNG1 signal (Fig. 1C). In parasites with retracted conoids, labeling is coincident with the RNG1 marker (Fig. 1D). TgSAS6L remains associated with the cytoskeleton following treatment with deoxycholate (DOC), which extracts much of the inner membrane complex-associated cytoskeleton and frees the subpellicular microtubules. In DOC-extracted samples, TgSAS6L labeling is at the apical tip of the conoid (Fig. 1E). Immunogold labeling of TgSAS6L-YFP parasites with an anti-GFP antibody indicates that the protein is located at the apex of the conoid, in a region consistent with the preconoidal rings (Fig. 1F).

### SAS6L-null Toxoplasma are less fit.

**Fig 2:**
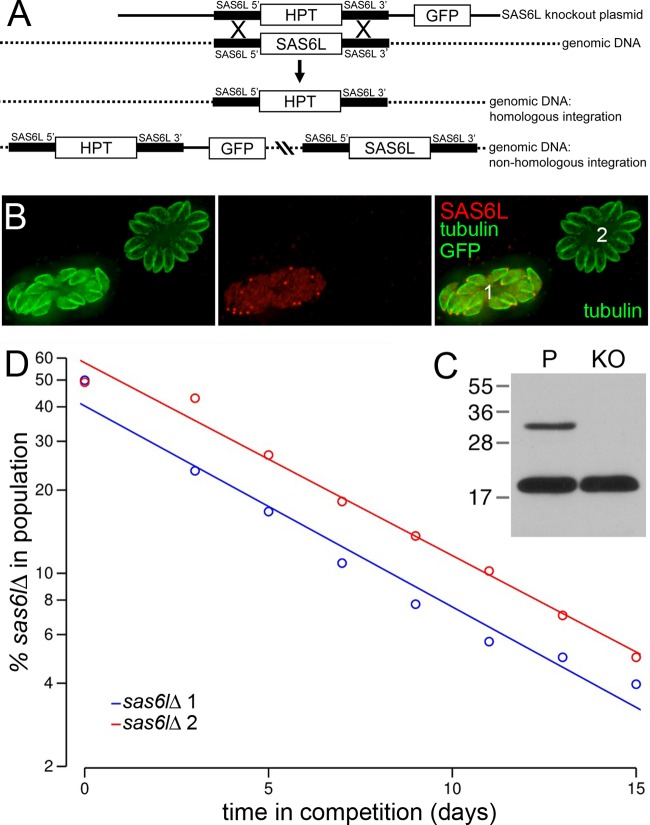
Loss of TgSAS6L reduces Toxoplasma fitness. (A) Knockouts were generated by homologous recombination of the HPT gene into the TgSAS6L locus. In the case that the vector integrates nonhomologously, both the GFP and HPT genes are integrated, generating fluorescent parasites. (B) *sas6l*Δ mutants were confirmed by loss of immunofluorescence labeling for TgSAS6L (red). All parasites (1 and 2) are labeled for tubulin (green). Nonhomologous integration of the knockout vector (parasite 1) leads to parasites that retain TgSAS6L and also have the GFP signal. (C) Immunoblotting with both anti-TgSAS6L and anti-ISP3 antisera also demonstrates the complete absence of TgSAS6L from *sas6l*Δ mutant knockout (KO) cells relative to the parental line (P). The values to the left are molecular sizes in kilodaltons. (D) Two independently derived *sas6l*-null parasite lines have reduced growth relative to that of GFP-expressing clones with a nonhomologous integration of the knockout vector.

We deleted the SAS6L gene from Toxoplasma tachyzoites by targeted integration of the HPT gene (Fig. 2A) and confirmed the knockout by the absence of SAS6L protein (Fig. 2B and C). Since there are no specific markers for the conoid fibers, we induced conoid extrusion and stained with a tubulin antibody to ascertain that the conoid is still present in null parasites (not shown). TgICMAP1 is a microtubule-associated protein that specifically localizes to the two intraconoid microtubules that run down the center of the conoid (64). We used an established expression construct (pmin-eGFP-TgICMAP1 [64]) to verify that the eGFP-TgICMAP1 fusion protein correctly localizes to the region of the intraconoid microtubules in the absence of SAS6L (not shown).

During creation of the *sas6l*Δ mutant lines, we also cloned lines that had nonhomologous integration of the knockout vector for use as a matched control in growth competition assays. We coinfected host cells with an equivalent number of each clonal line and followed the relative rates of replication and growth over serial passage by flow cytometry (Fig. 2D). Two independent knockout lines were outcompeted by a matched control at a rate of 0.07 day^−1^, demonstrating a reduction in fitness. The reduced *sas6l*Δ mutant parasite growth observed may reflect a specific defect in a number of cellular processes required for invasion and intracellular growth (for example, motility, attachment, invasion, doubling time, replication fidelity, egress, and extracellular viability). In order to assess specific defects in these processes, we measured gliding motility, performed plaque formation assays (for invasion and extracellular survival), assessed Ca^2+^ ionophore-mediated egress, and quantified replication rates and the frequency of replication defects. Within the resolution of these assays, no significant differences between two independent *sas6l*-null lines and the matched control were detected (data not shown). However, the expected size of a specific defect in assays such as the plaque assay (∼10%, given the rate of outcompetition) was below the assay's resolving power because of the relative variation between biological replicates. Hence, it is likely that the direct fitness assay detected a defect that was below the sensitivity of the other assays in our experiments.

### SAS6L is an ancient protein found in diverse simple eukaryotes.

**Fig 3:**
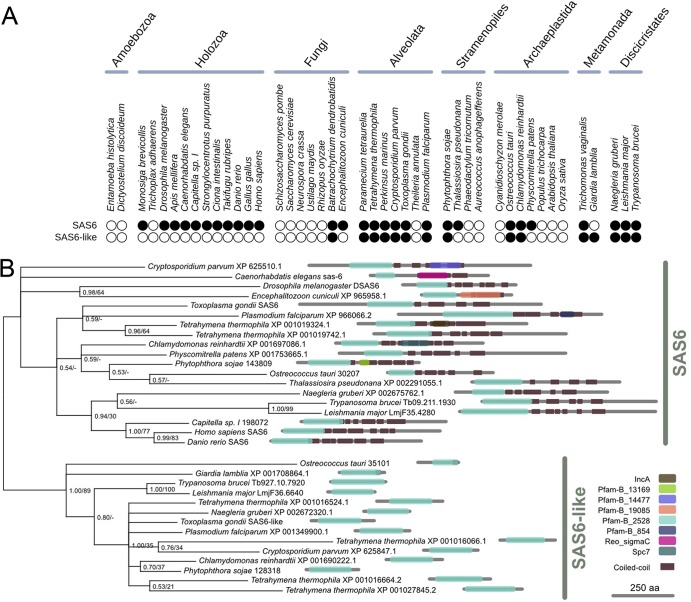
SAS-6 and SAS6L proteins are found in a subset of organisms that form cilia or flagella. (A) Presence (filled circles) or absence (empty circles) of identifiable homologues of SAS-6 and SAS6L in the predicted proteomes of 44 diverse eukaryotes. SAS6L protein is present in most organisms that also possess SAS-6 but absent from the Holozoa. (B) The SAS-6 and SAS6L protein families share a conserved domain of ∼200 aa but can be robustly separated by phylogenetic analysis and have distinct architectures. A Bayesian phylogeny of the conserved domain is shown with topology support from Bayesian posterior probabilities and maximum likelihood bootstrap replicates (PP/BS). Architectures show predictions of Pfam-A and -B domains (89) and coiled-coil regions (90).

Similarity searches suggested the presence of SAS6L homologs in diverse eukaryotes. All SAS6L proteins contain an ∼200-amino-acid (aa) conserved domain that is shared with SAS-6 homologs (see Fig. S1 in the supplemental material) and includes the ∼50-aa PISA motif previously described in SAS-6 (42). This domain is described by the automatically generated Pfam-B domain, Pfam_B_2528. We used Pfam_B_2528 to identify proteins of the SAS-6/SAS6L family encoded by the genomes of 29 of 44 diverse eukaryotes (Fig. 3A). These proteins share an average of 24% identity (46% similarity) across the conserved domain. A similar set was defined by an alternative approach based on clustering by BLASTp score (data not shown).

Phylogenetic analysis based on the conserved domain robustly supported the existence of two subfamilies comprising SAS-6 and SAS6L (Fig. 3B). Genes encoding SAS-6 are found in the genomes of eukaryotes that are known to build a centriole or basal body and also in the microsporidian Encephalitozoon cuniculi and Ostreococcus tauri as previously described (42–45, 60). With the exception of Giardia lamblia, which lacks an identifiable SAS-6 ortholog (60), SAS6L is found only in lineages that also contain SAS-6 (Fig. 3A). In addition to Toxoplasma, SAS6L is found in multiple protistan lineages (including other apicomplexans, such as Plasmodium and Cryptosporidium species). However, SAS6L appears to be absent from metazoan and choanoflagellate genomes (Holozoa). The presence of SAS6L in a basal flagellate fungus (Batrachochytrium dendrobatidis) indicates that the absence of SAS6L from animals is likely the result of a specific loss near the root of Holozoa.

Although only the conserved domain was used to define the phylogenetic families, the SAS-6 and SAS6L proteins have clearly distinct architectures (Fig. 3B). SAS-6 sequences are generally ∼600 aa in length and comprise the conserved domain near the N terminus, followed by ∼300 aa of sequence rich in predicted coiled coils. In contrast, SAS6L sequences contain little sequence outside the conserved domain and appear to lack the C-terminal tail entirely. The conserved domain essentially matches the globular head domain of SAS-6, the structure of which has been solved (50, 51). Homology modeling suggests that SAS6L proteins can adopt structures very similar to those seen in the head of SAS-6 (see Fig. S2 in the supplemental material). The apicomplexan SAS-6 proteins are unusual in possessing long N-terminal extensions before the conserved domain (Fig. 3B).

### TbSAS6L localizes to the axonemal basal plate in trypanosomes.

**Fig 4:**
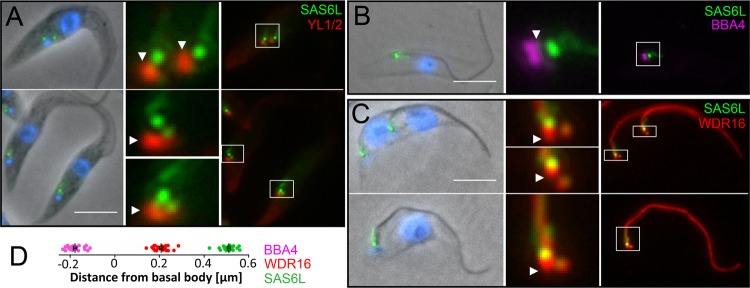
TbSAS6L-YFP localizes to a prominent focus near the base of the flagellum in T. brucei. (A) TbSAS6L-YFP (green) is distal to the basal body, which is marked by tyrosinated α-tubulin (YL1/2, red, arrowheads). A lower-intensity SAS6L-YFP signal is also associated with the developing probasal body. SAS6L-YFP is also distal to the basal-body markers BBA4 (B) and WDR16 (C) (arrowheads). (D) Distance measurements in relation to YL1/2 indicate that SAS6L-YFP localizes to a region near the axoneme basal plate (20 measurements for each marker). Scale bars, 5 μm.

Antiserum against TgSAS6L labels a spot at the apex of the related parasite Neospora caninum (not shown). Unfortunately, our antiserum does not label SAS6L proteins in more distantly related organisms, such as Tetrahymena thermophila (data not shown). Since the majority of SAS6L-producing eukaryotes do not have a conoid, we tested the localization of a SAS6L ortholog in a distantly related organism. In Trypanosoma brucei trypomastigotes constitutively expressing TbSAS6L-YFP from the endogenous locus, TbSAS6L-YFP localizes near the base of the flagellum (Fig. 4A to C). Label appears as one or two prominent dots, depending on the cell cycle stage, and there is also weaker labeling in the proximal region of the axoneme, in the region of the flagellar pocket collar. TbSAS6L-YFP labeling is distal to markers of the proximal (BBA4), middle (tyrosinated α-tubulin, YL1/2), and distal (WDR16) parts of the basal body. Quantification of the location of TbSAS6L-YFP relative to these markers indicates that this protein is located in a fixed position within the axoneme (Fig. 4D), in a region consistent with the location of the basal plate (the area where the central-pair microtubules of a flagellar axoneme are nucleated). Ablation of TbSAS6L by RNA interference does not alter the overall morphology of the flagellum, as viewed by EM, suggesting that SAS6L has a nonessential role in flagellum formation (not shown). This is not entirely surprising, given the plasticity of flagellum formation, but it is also, of course, extremely difficult to detect subtle alterations in this complex structure.

### Overexpression of SAS6L-YFP causes ectopic filament formation.

**Fig 5:**
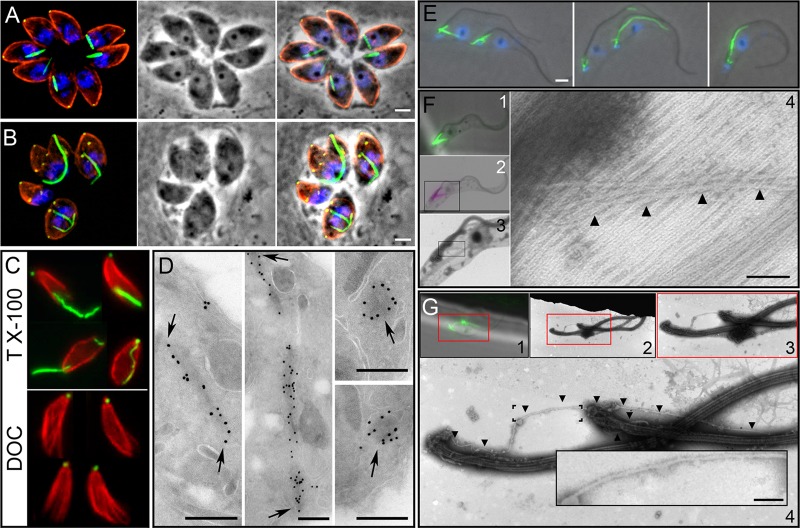
Overexpression of SAS6L-YFP leads to filament formation. (A) Intracellular Toxoplasma tachyzoites show prominent TgSAS6L-YFP filaments as follows: Toxoplasma tubulin, red; SAS6L-YFP, green; DNA, blue. (B) TgSAS6L-YFP filaments appear to interfere with the completion of division by some tachyzoites. (C) SAS6L-YFP filaments are insensitive to 1% Triton X-100 (TX-100), but 10 mg/ml DOC causes filament depolymerization although apical SAS6L is retained. (D) EM of immunogold-labeled cryosections of Toxoplasma overexpressing TgSAS6L-YFP show electron-dense structures of variable width within the cytoplasm. (E) Overexpression of the TbSAS6L protein in trypanosomes also leads to ectopic filaments. (F) Correlative light microscopy and EM of TbSAS6L-YFP filaments in trypanosomes (arrowheads). The same cell cytoskeleton is shown by phase-contrast microscopy (F1) and negatively stained transmission EM (F2 to -4). (G) Correlative microscopy of Ca^2+^-treated cytoskeleton preparations shows that TbSAS6L-YFP filaments are resistant to Ca^2+^ (arrows). Scale bars: A and B, 2 μm; D, 250 nm; E, 2 μm; F4, 200 nm; G4 inset, 200 nm.

To investigate the *in vivo* properties of SAS6L, we expressed tagged SAS6L in both Toxoplasma and Trypanosoma over endogenous protein. In Toxoplasma, when TgSAS6L-YFP expression was driven from the α1-tubulin promoter, excess protein assembled into filaments in a subset of the sibling Toxoplasma parasites within a single parasitophorous vacuole (Fig. 5A). Filaments sometimes deformed the parasite's shape and may interfere with cell division, as in the parasites shown in Fig. 5B, where a large filament extends between daughters that have begun a new round of replication without completing scission. TgSAS6L-YFP filaments form when intracellular parasites are treated with oryzalin, which inhibits microtubule polymerization (not shown). Ectopic TgSAS6L-YFP filaments are retained after extraction with 1% Triton X-100 but are sensitive to 10 mg/ml DOC, which removed TgSAS6L-YFP filaments without disrupting the apical TgSAS6L signal (Fig. 5C). Immunogold staining of EM cryosections of whole cells showed that TgSAS6L-YFP was associated with continuous electron-dense structures in the cytoplasm (Fig. 5D).

Overexpression of TbSAS6L-YFP in trypanosomes also resulted in the accumulation of ectopic filaments (Fig. 5E). The formation of these structures has no effect on the morphology or duplication time of the cells (data not shown). The filaments are insensitive to extraction with nonionic detergent (Nonidet P-40) and are not labeled with anti-tubulin antibodies (not shown), indicating that they are independent of microtubules. TbSAS6L-YFP filaments are insensitive to treatment with Ca^2+^, which depolymerizes T. brucei subpellicular microtubules (Fig. 5G). Correlative microscopy of detergent-extracted trypomastigotes was used to unambiguously determine the ultrastructure of TbSAS6L-YFP filaments by first identifying filaments by fluorescence microscopy and subsequently imaging them by EM (Fig. 5F and G). TbSAS6L filaments are ∼20 nm wide but have a structure distinct from that of microtubules (Fig. 5G).

## DISCUSSION

We describe a novel conserved protein that is related to the centriole protein SAS-6, which we have hence named the SAS-6-like (SAS6L) protein. The existence of SAS6L produced by diverse organisms suggests that this protein appeared early in eukaryote evolution and most likely before the last common ancestor of all extant eukaryotes (65). SAS6L is found in simple eukaryotes that contain SAS-6 and centrioles/basal bodies, but the protein has been lost from the holozoan lineage. SAS6L proteins consist of a conserved domain that also comprises the N-terminal head domain of SAS-6. Recent data have shown that SAS-6 forms homodimers through interactions between their coiled-coil tail domains and that these homodimers interact via other parts of the head domain to form higher-order structures (in particular, rings) (50, 51). Since SAS6L proteins lack a SAS-6 tail, they cannot dimerize by this means. However, the opposite face of the protein is available for interaction and is predicted to have a structure very similar to that of SAS-6. The observation that overexpression of SAS6L proteins induces SAS6L filament formation indicates that SAS6L proteins do, indeed, have the ability to form higher-order structures. However, the distinct localization of SAS-6 and SAS6L proteins in Toxoplasma and Trypanosoma suggests that these proteins do not form heterodimers *in vivo*. The decreased stability of the ectopic SAS6L filaments after DOC extraction suggests that additional interactions stabilize the native preconoidal ring structure and do not occur in the cytoplasmic filaments.

Consistent with its location in other diverse eukaryotes, SAS-6 localizes to the basal body in Trypanosoma and the centriole Toxoplasma tachyzoites. In contrast, SAS6L localizes to the region of the basal plate in Trypanosoma brucei—the electron-dense region at the distal end of the transition zone from which the central-pair microtubules are nucleated. Unlike T. brucei (where flagella are present in all life cycle forms), only the microgamete forms of Toxoplasma or other apicomplexan organisms build flagella (24, 32, 66–69). Comparative genomics indicate that the Toxoplasma and Plasmodium genomes lack a surprising number of conserved basal-body components (60), and while studies of Plasmodium microgamete development show *de novo* formation of basal bodies (66), it is unclear whether Toxoplasma microgamete basal bodies form *de novo* or if the atypical centriole found in the asexual tachyzoite stage serves as a template for basal-body formation. We anticipate that SAS6L will also localize to the basal plate region in Toxoplasma microgametes, but because of the difficulty in obtaining this stage from cat intestinal epithelium, we cannot verify this prediction. In agreement with this hypothesis, there is evidence of upregulation of the Plasmodium falciparum ortholog of SAS6L (PF3D7_1316400) in gametocyte development (70).

The Toxoplasma tachyzoite stage causes most of the pathology associated with parasite infection. Despite lacking flagella, tachyzoites are motile and employ an unusual substrate-dependent gliding motility that requires actin and myosin (71–73). The apical region of tachyzoites is vital to host cell invasion and contains specialized secretory organelles (micronemes and rhoptries) and a complex and unusual organization of the microtubule cytoskeleton (28, 39, 74, 75). The elongated cell shape of tachyzoites is maintained by a corset of 22 subpellicular microtubules that radiate out of the APR. An additional tubulin-based structure, the conoid, can be extended through the APR or retracted into it. In intracellular parasites, the conoid is retracted and immotile, while in extracellular parasites, the conoid can extend and retract as tachyzoites glide and invade host cells (36). Extension of the conoid is associated with the elongation and narrowing of the apical end of tachyzoites, and it is believed that the conoid has a mechanical role in parasite invasion of host cells.

**Fig 6:**
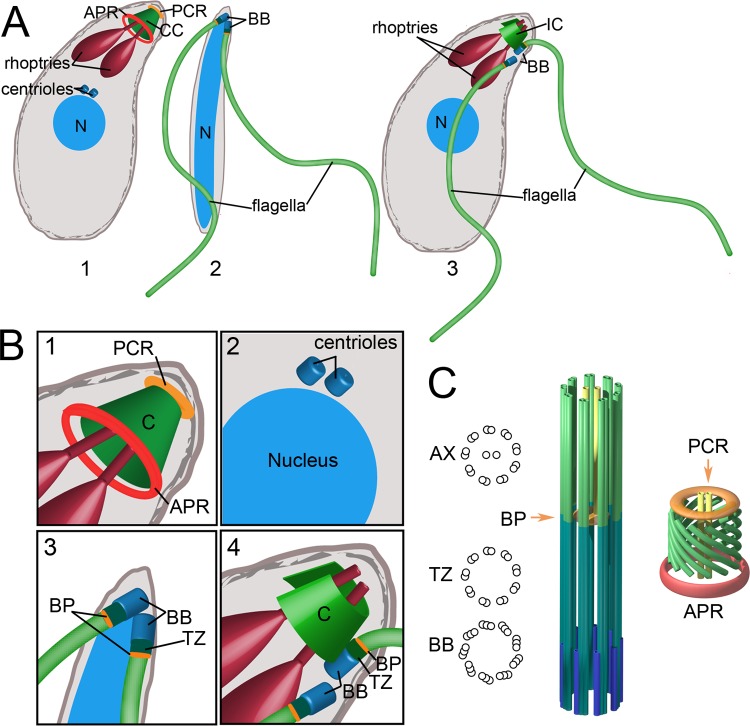
The Toxoplasma conoid incorporates flagellar components, and its appearance and that of flagella are mutually exclusive during the parasite life cycle. (A) Toxoplasma tachyzoites (part 1) have juxtanuclear centrioles and a specialized apical region used to invade host cells. Rhoptries (red) secrete components used during invasion, while the conoid (green) can extend from the APR to cause elongation of this region. Toxoplasma tachyzoites have a closed conoid (CC) that is topped by preconoidal rings (PCR), the location of SAS6L (orange). Toxoplasma microgametes (part 2) lack a conoid but have apically located basal bodies (BB) that template two flagella that extend toward the posterior. Alveolate organisms such as Colpodella and Rastrimonas (part 3) contain rhoptry-like secretory organelles (red) and an incomplete conoid (IC) structure consisting of a set of interlinked microtubules at the cell apex. They build flagella from adjacent basal bodies (BB) but lack the conoid-associated APR structure observed in coccidians. (B) The Toxoplasma tachyzoite conoid consists of 10 to 14 tubulin-containing filaments that spiral to form a closed cone that is narrower at the apex than at the base (box 1). The conoid may be extended from or retracted into the APR, which also serves to nucleate 22 subpellicular microtubules (not shown). The SAS6L protein is located at the extreme tip of the conoid (orange) in the region of the PCR. The juxtanuclear centrioles (box 2) are located at a distance from the conoid in tachyzoites. Toxoplasma microgametes (box 3) have apical centrioles that build a transition zone (TZ) and template flagellar axonemes. We predict that SAS6L localizes to the basal plate (BP) in this stage (orange). Alveolates such as Colpodella (box 4) have apical secretory organelles (red) and an incomplete conoid (IC) structure (green) that does not fully encircle the rhoptry necks and may be as simple as a set of four to six interlinked microtubules. Adjacent apical centrioles build a transition zone (TZ) to template flagellar axonemes with a basal-plate (BP) structure. These organisms lack the conoid-associated APR structure observed in Toxoplasma. (C, left) The basal body (BB) templates the flagellar axoneme (AX). The transition from the triplet microtubule organization of the basal body to the doublet microtubule organization of the axoneme occurs at the basal plate (BP), which is also where the central-pair microtubules of the axoneme begin. SAS6L in trypanosomes localizes to the region of the flagellar plate (orange). (C, right) The Toxoplasma conoid is formed from 10 to 14 C-shaped and spiraling tubulin filaments. Two 13-protofilament microtubules extend through the center of the conoid, reminiscent of the central-pair microtubules of the axoneme. The conoid ends in two preconoidal rings, and Toxoplasma SAS6L localizes to this region (orange). The conoid can be extended beyond or retracted into the APR (red), which serves as an MTOC for the subpellicular microtubules.

Apicomplexans share ancestry with ciliates and dinoflagellates; collectively, these organisms are classified as alveolates (76, 77). It is likely that the last common ancestor of apicomplexans and dinoflagellates had an open-sided “incomplete” conoid that was modified into a closed conoid in apicomplexans and lost from dinoflagellates (77, 78). Within the Apicomplexa, the conoid was again lost from noncoccidian lineages. Although “true” conoid structures are found only in coccidian apicomplexans, alveolates such as Colpodella vorax and Rastrimonas subtilis (previously known as Cryptophagus subtilis) have “pseudoconoid” or “incomplete conoid” structures consisting of a set of 5 to 14 interlinked microtubules located at the cell apex but lack the adjacent APR structure observed in coccidians (78–81). Colpodella and Rastrimonas also contain apical secretory organelles reminiscent of apicomplexan micronemes and rhoptries that are used along with a pseudoconoid to partially or completely invade other unicellular protists. Colpodella attaches to and aspirates the cytoplasmic contents of unicellular flagellates (76, 78, 80, 81), while Rastrimonas is an intracellular parasite of unicellular algae (79, 80). The precise phylogenetic position of Colpodella and Rastrimonas is unclear, as these alveolates have been variously described as dinoflagellates, as early-branching apicomplexans, as Perkinsozoa, or within sister lineages of dinoflagellates or apicomplexans (76, 78, 80, 81). What is clear is that these organisms are poised between predator and parasite states and provide tantalizing clues to the evolution of the intracellular apicomplexan lifestyle. Both Colpodella and Rastrimonas simultaneously build a pseudoconoid structure and adjacent flagella (Fig. 6A and B). In contrast, the flagellar axoneme and “true” conoid are mutually exclusive structures in coccidian parasites; microgametes have apically located basal bodies that template flagellar axonemes, while asexual proliferative-stage coccidians have juxtanuclear centrioles and apical conoid structures (Fig. 6A and B). This study describes SAS6L, a novel conserved protein that is located in the region of the basal plate of the axoneme in T. brucei and above the conoid in Toxoplasma tachyzoites, a life cycle stage that lacks flagella (Fig. 6C). Rastrimonas absorbs its flagella during intracellular growth, and this feature may have become more pronounced during the evolution of apicomplexan parasites, ultimately leading to the loss of flagella in all stages other than microgametes. Our results emphasize this relationship between the conoid and flagellum, providing molecular evidence of a link between these organelles.

The observations presented here are reinforced by recent work on the basal-body-associated protein SFA (striated fiber assemblin) in Toxoplasma. SFA proteins were first described in green algae, where flagellar basal bodies are embedded in a “cage” of flagellar rootlet fibers. Rootlets consist of distinct populations of centrin-containing contractile fibers (82), sinister fibers (83), and SFA-containing noncontractile striated fibers (84, 85). Genes for SFA homologs are found in apicomplexan genomes (86, 87). Remarkably, two SFA proteins (TgSFA2 and TgSFA3) form a filament that links the upper edge of the conoid to the juxtanuclear centrioles during tachyzoite replication (88). The SFAs appear shortly after centriole duplication (87), colocalizing in a fiber-like structure that emerges from the centrioles and extends apically to tether the conoid and APR to the centrioles (88). Loss of either SFA protein inhibits parasite replication by blocking the formation of daughter APRs and the subsequent construction of daughter buds. Nuclear division is not inhibited, and multiple nuclei accumulate in these aberrant parasites. These results suggest that although the asexual stages of apicomplexans have dispensed with building flagella, they retain SFAs to ensure the reliable inheritance of apical organelles.

Given the observation that the SFAs and SAS6L are retained in aflagellate tachyzoites, an intriguing possibility is that SAS6L is one of the components that tether the SFA filament to the conoid. SAS6L cannot be the sole factor anchoring SFA to the apex, as our results indicate that SAS6L is not essential (although its loss is associated with a fitness defect) while loss of either SFA2 or SFA3 leads to a lethal arrest at the time of daughter cell budding. In light of our data on SAS6L and the observations on SFA2 and SFA3, we propose that the complete conoid evolved from an ancestral pseudoconoid in part by the incorporation of components from a vestigial apical flagellar apparatus. These components may have been from flagellum-associated structures, such as flagellar rootlets (as seen for the small portion of Trypanosoma SAS6L that is associated with the flagellar collar region), or could represent the direct subsumption of the flagellum into the conoid. The former premise suggests that flagellar components are retained because they are required for an essential cellular process, such as replication. In this context, SAS6L may have been retained along with the basal-body-associated SFA proteins in order to coordinate organelle inheritance in aflagellar tachyzoites (88). The latter hypothesis implies direct homology between the central-pair microtubules of the axoneme and the microtubule pair at the center of the conoid and suggests that other orthologs of basal-plate and central-apparatus components may play a role in invasion or replication by coccidia.

## Supplementary Material

Supplemental material
