# Supplementary material for: A SAS-6-Like Protein Suggests that the Toxoplasma Conoid Complex Evolved from Flagellar Components
Source: Eukaryot Cell. 2013 Jul;12(7):1009–19. doi: 10.1128/EC.00096-13 (PMC3697468; doi:10.1128/EC.00096-13)
Supplement: Supplemental material [file supp_12_7_1009__index.html]

Supplemental material 

# A SAS-6-Like Protein Suggests that the Toxoplasma Conoid Complex Evolved from Flagellar Components

## 

**Files in this Data Supplement:**

- Supplemental file 1 -

  Alignment of the conserved domain of SAS6 and SAS6L proteins (Fig. S1) and structures of SAS6L proteins predicted by homology modeling (Fig. S2).

  PDF, 3.5M
