# Supplementary material for: A SAS-6-Like Protein Suggests that the Toxoplasma Conoid Complex Evolved from Flagellar Components
Source: Eukaryot Cell. 2013 Jul;12(7):1009–19. doi: 10.1128/EC.00096-13 (PMC3697468; doi:10.1128/EC.00096-13)
Supplement: Supplemental material [file EC.00096-13_zek999094124so1.pdf]

•Supplemental Figure 1

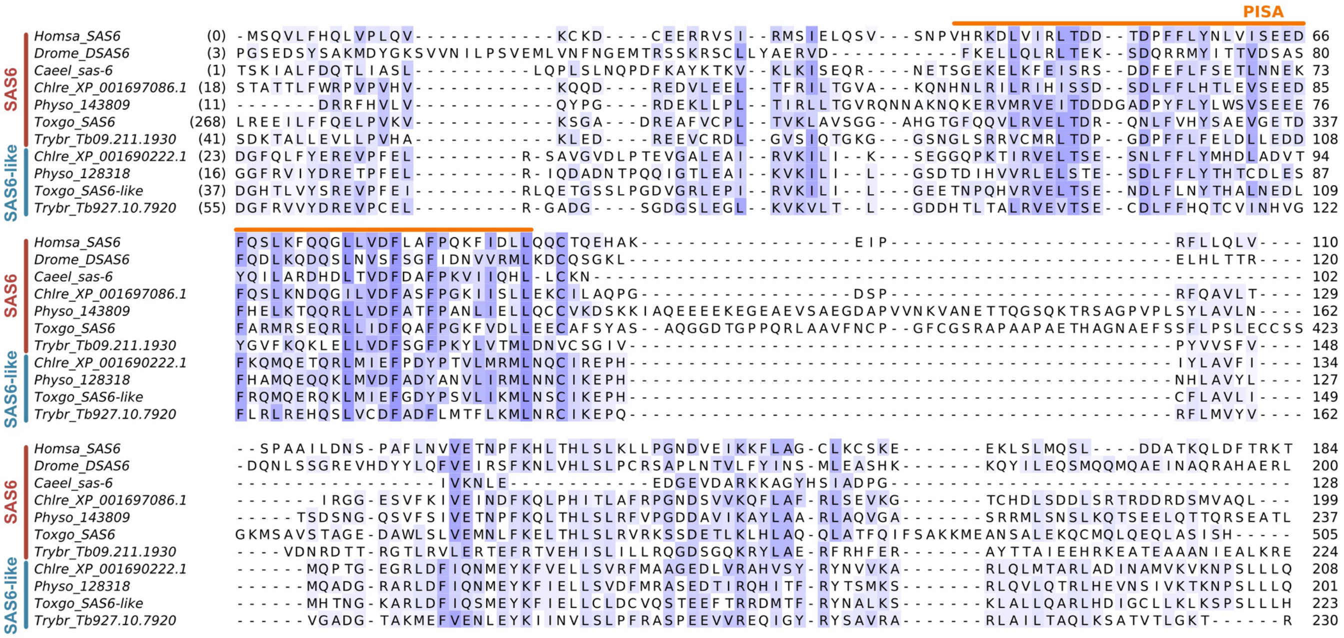

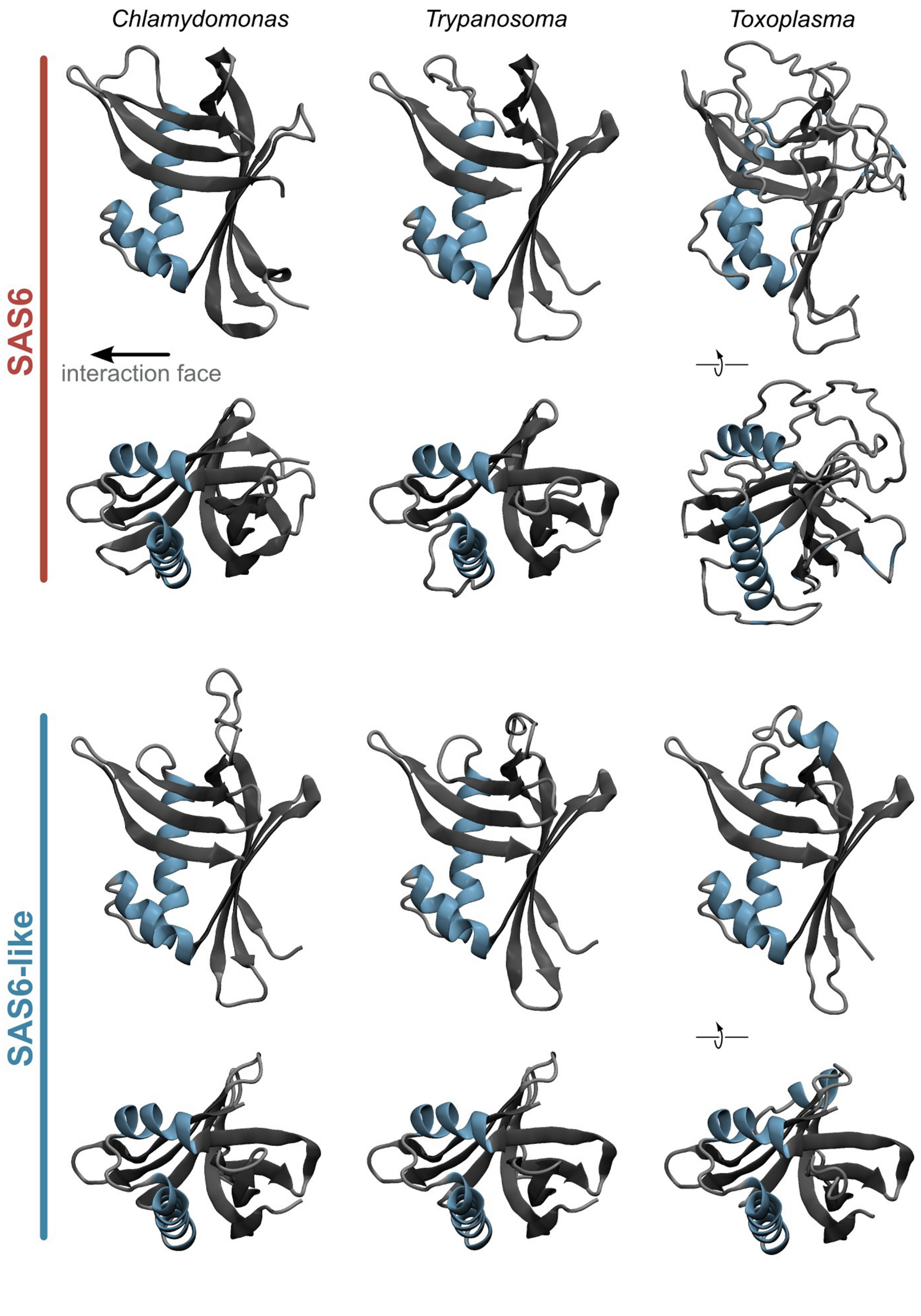
Chlamydomonas
Trypanosoma
Toxoplasma
SAS6
interaction face
SAS6-like

Figure S1: Alignment of the conserved domain of SAS6 and SAS6-like proteins. Domains from 7 representative species are shown: Caeel: *Caenorhabdatis elegans*; Chlre: *Chlamydomonas reinhardtii*; Drome: *Drosophila melanogaster*; Homsa: *Homo sapiens*; Physo: *Phytophthora sojae*; Toxgo: *Toxoplasma gondii*; Trybr: *Trypanosoma brucei*. Individual residues are shaded according to their degree of conservation. Orange bar highlights the position of the "PISA" motif previously described for SAS-6 proteins (42). Numbers in brackets at N-terminus indicate preceding residues not shown in alignment.

Figure S2: Structures of SAS6L proteins predicted by homology modeling. Conserved head domain structures from the SAS-6 and SAS6L homologues present in *Toxoplasma gondii* and *Trypanosoma brucei* and also the *Chlamydomonas reinhardtii* SAS6L protein were predicted by template-based modeling based on the solved structure of the *Chlamydomonas reinhardtii* SAS-6 head domain (Protein Data Bank entry: 3Q0Y) (50). Modeling was performed using the program I TASSER (91) and models visualised with PyMOL (http://pymol.org/). Two orthogonal views are shown for each protein. The direction of the interaction face through which SAS-6 dimers can multimerize, is also indicated.
